# Impact of Preoperative Neutrophil to Prealbumin Ratio Index (NPRI) on Short-Term Complications and Long-Term Prognosis in Patients Undergoing Laparoscopic Radical Surgery for Colorectal Cancer

**DOI:** 10.1155/2024/4465592

**Published:** 2024-04-26

**Authors:** Wenliang Jiang, Yong Xia, Yujun Liu, Shaoqi Cheng, Wenya Wang, Zhenghui Guan, Hongmei Dou, Changhe Zhang, Honggang Wang

**Affiliations:** ^1^Postgraduate Training Base of Dalian Medical University (Taizhou People's Hospital), 366 Taihu Road, Taizhou, Jiangsu, China; ^2^Department of General Surgery, Gaoyou People's Hospital, 10 Dongyuan Road, Gaoyou City, Jiangsu Province, China; ^3^Department of General Surgery, The Affiliated Taizhou People's Hospital of Nanjing Medical University, Taizhou School of Clinical Medicine, Nanjing Medical University, 366 Taihu Road, Taizhou, Jiangsu, China; ^4^Department of Operating Room, The Affiliated Taizhou People's Hospital of Nanjing Medical University, Taizhou School of Clinical Medicine, Nanjing Medical University, 366 Taihu Road, Taizhou, Jiangsu, China

## Abstract

**Objective:**

This study aims to evaluate the impact and predictive value of the preoperative NPRI on short-term complications and long-term prognosis in patients undergoing laparoscopic radical surgery for colorectal cCancer (CRC).

**Methods:**

A total of 302 eligible CRC patients were included, assessing five inflammation—and nutrition-related markers and various clinical features for their predictive impact on postoperative outcomes. Emphasis was on the novel indicator NPRI to elucidate its prognostic and predictive value for perioperative risks.

**Results:**

Multivariate logistic regression analysis identified a history of abdominal surgery, prolonged surgical duration, CEA levels ≥5 ng/mL, and NPRI ≥ 3.94 × 10^−2^ as independent risk factors for postoperative complications in CRC patients. The Clavien–-Dindo complication grading system highlighted the close association between preoperative NPRI and both common and severe complications. Multivariate analysis also identified a history of abdominal surgery, tumor diameter ≥5 cm, poorly differentiated or undifferentiated tumors, and NPRI ≥ 2.87 × 10^−2^ as independent risk factors for shortened overall survival (OS). Additionally, a history of abdominal surgery, tumor maximum diameter ≥5 cm, tumor differentiation as poor/undifferentiated, NPRI ≥ 2.87 × 10^−2^, and TNM Stage III were determined as independent risk factors for shortened disease-free survival (DFS). Survival curve results showed significantly higher 5-year OS and DFS in the low NPRI group compared to the high NPRI group. The incorporation of NPRI into nomograms for OS and DFS, validated through calibration and decision curve analyses, attested to the excellent accuracy and practicality of these models.

**Conclusion:**

Preoperative NPRI independently predicts short-term complications and long-term prognosis in patients undergoing laparoscopic colorectal cancer surgery, enhancing predictive accuracy when incorporated into nomograms for patient survival.

## 1. Introduction

In a global context, colorectal cancer (CRC) stands as the third most prevalent malignancy, trailing only lung cancer, and serves as a common cause of cancer-related mortality [1]. Surgical resection is currently the primary treatment for Stages I and II CRC, proven to be the sole curative approach. Since the 1990s, laparoscopic surgery has emerged as an effective alternative to open surgery, showcasing advancements in achieving excellent oncological radicality while enhancing the protection of the pelvic nerve plexus [2, 3]. Notably, laparoscopic surgery is associated with shorter hospital stays, reduced rates of wound infections, alleviated pain, and faster recovery compared to open surgery [4]. Despite significant improvements in surgical techniques, perioperative management, and multidisciplinary treatment, there is limited progress in the prognosis, survival, and prevention of recurrence and metastasis for some patients. Furthermore, complications such as anastomotic leakage, intestinal obstruction, and bleeding after laparoscopic CRC radical surgery continue to pose a significant risk, significantly impacting both short-term quality of life and long-term oncological outcomes [5]. Therefore, the development of optimal biomarkers for predicting postoperative complications and adverse outcomes following laparoscopic CRC radical surgery holds paramount importance in clinical practice.

The established connection between inflammation and cancer involves the host's immune response to tumors and the release of inflammatory chemokines and cytokines by tumor-associated leukocytes and cancer cells, influencing various aspects of tumor biology [6]. Recent reports confirm that inflammation markers can serve as predictors of postoperative complications and prognosis in cancer patients. These markers include neutrophils, lymphocytes, platelets, monocytes, and combinations thereof, such as the platelet-to-lymphocyte ratio (PLR) and the neutrophil-to-lymphocyte ratio (NLR) [7, 8]. Lymphocytes play a crucial role in inhibiting cancer cell proliferation, and their significant reduction weakens immune responses, particularly in advanced cancer [9]. Platelets, induced by tumor cells, release growth factors, promoting angiogenesis and tumor growth [10]. Neutrophils, part of the innate immune system, promote cancer development by releasing the extracellular matrix and inflammatory factors in the tumor microenvironment [11]. Malnutrition, common in cancer patients, significantly impacts prognosis [12]. Preoperative malnutrition in CRC patients increases the risk of postoperative complications and affects prognosis [13]. Serum albumin and prealbumin are common indicators for assessing nutritional status, exhibiting antioxidant and anti-inflammatory effects. Albumin is the most abundant plasma protein found in humans. Lower levels of albumin indicate malnutrition or are associated with the inflammatory processes that inhibit albumin production or increase albumin consumption [14]. On the other hand, prealbumin, a nonglycosylated plasma protein synthesized in the liver, plays a role in transporting thyroid hormones and vitamin A [15]. It demonstrates good sensitivity and specificity to changes in protein synthesis and breakdown metabolism, proving to be more sensitive than serum albumin. Consequently, it can more effectively assess patients' protein consumption and nutritional status [16].

The combination of inflammation markers and nutritional assessment aids in predicting postoperative complications and prognosis in cancer patients. Recent research identifies the neutrophil-to-albumin ratio (NAR) as an independent influencing factor for the mortality of patients with pancreatic [17] and rectal cancer [18], demonstrating high sensitivity. The predictive value of indicators such as the C-reactive protein-to-albumin ratio, the fibrinogen-to-albumin ratio, and others in the treatment of malignant tumors has also been successively reported [19, 20]. Currently, the preoperative NPRI has only been reported to be associated with the prognosis of curative resection of intrahepatic cholangiocarcinoma [21], but its impact on postoperative complications and long-term prognosis in patients undergoing laparoscopic radical resection for colorectal cancer has not been reported. This study aims to investigate the relationship between preoperative NPRI and short-term complications and long-term prognosis in patients undergoing laparoscopic radical CRC surgery. Additionally, the study seeks to construct line graph prediction models for 1-, 3-, and 5-year OS and DFS in CRC following laparoscopic radical surgery, providing a theoretical basis for improving perioperative treatment.

## 2. Materials and Methods

### 2.1. Study Population

A total of 302 cases meeting the inclusion criteria were retrieved from the Hospital Information System (HIS) of Taizhou People's Hospital, who had undergone surgical treatment in the Department of Gastrointestinal Surgery between June 2015 and June 2017.

#### 2.1.1. Inclusion Criteria

1. Preoperative diagnosis of CRC confirmed by postoperative pathology;
2. TNM stage of CRC as stages I–III;
3. Blood samples obtained within 1 week prior to surgery to reflect preoperative baseline levels;
4. Elective laparoscopic surgery performed with complete resection of the tumor, excluding emergency cases to avoid confounding factors related to surgical approach influencing the interpretation of results;
5. Complete clinical and pathological data available to ensure data quality;
6. Postoperative follow-up data available for evaluation of long-term prognosis;
7. Approval obtained from the Clinical Research Ethics Committee of Taizhou People's Hospital.

#### 2.1.2. Exclusion Criteria

1. Incomplete clinical data, such as coagulation function and hepatorenal function, to ensure inclusion of patients with complete baseline data;
2. Distant metastasis or concomitant other malignancies to reduce the influence of other diseases;
3. Concomitant autoimmune diseases, as they may affect inflammatory and nutritional status;
4. Preoperative neoadjuvant chemoradiotherapy, as it may alter the patient's inflammatory and nutritional status affecting baseline levels of inflammatory markers;
5. Secondary colorectal cancer to ensure the study population consists of primary colorectal cancer patients;
6. Perioperative mortality, as these patients lack data on postoperative complications and long-term survival;
7. Severe systemic infectious symptoms, as infections can lead to changes in inflammatory and nutritional status that can affect levels of inflammatory markers;
8. Loss to follow-up after surgery to ensure complete follow-up data.

### 2.2. Study Methods

#### 2.2.1. Data Collection

Patient medical records were collected, including basic information, medical history, preoperative laboratory results, postoperative pathology, TNM stage of AJCC eighth edition [22], surgical details, complications, and follow-up outcomes. BMI, NPRI, NLR, NAR, and platelet-to-albumin ratio (PAR) values were calculated.

#### 2.2.2. Sample Collection Method

Within 1 week prior to surgery, 3–5 mL of venous blood was drawn from the cubital vein of CRC patients and sent to the laboratory department of Taizhou People's Hospital for relevant testing. Neutrophils, lymphocytes, platelets, prealbumin, albumin, hemoglobin, and carcinoembryonic antigen (CEA) were measured as part of routine preoperative testing. Complete blood cell counts were analyzed using the Mindray BC-5000 fully automated hematology analyzer. Albumin and prealbumin levels were measured by the Beckman Coulter UniCel DxC 800 Synchron fully automated biochemistry analyzer, while CEA was detected using the Roche Cobas 6000 fully automated electrochemiluminescence immunoassay analyzer. The respective companies provided corresponding quality control and calibration materials.

#### 2.2.3. Grouping Method

Clinical factors were categorized, and patients were grouped based on optimal cutoff values for NPRI, NLR, PLR, NAR, and PAR. Complications were classified using the Clavien–Dindo system.

#### 2.2.4. Postoperative Follow-Up

A total of 336 eligible patients from Taizhou People's Hospital were enrolled in this study. The follow-up period ranged from 10 to 65 months (mean: 55.23 ± 9.07 months, median: 60 months) until June 2023. Thirty-four patients were lost to follow-up, resulting in data successfully collected from 302 patients (attrition rate: 10.12%). Follow-up assessments were conducted at scheduled intervals over 5 years, with examinations at 1, 3, 6, 9, and 12 months after the first postoperative year, followed by evaluations every 6 months thereafter. The follow-up concluded upon patient death, encompassing examinations of patients' quality of life, survival status, and recurrence.

### 2.3. Statistical Methods

Statistical analyses were performed using SPSS 17.0 and R software (version 4.2.1). Categorical data were analyzed using chi-square or Fisher's exact tests, and parametric data were assessed with *t*-tests. Non-normally distributed continuous data were presented using median values. ROC curves determined optimal cutoff values for continuous variables predicting complications and survival outcomes. Logistic regression analysis and univariate and multivariate Cox regression models were employed to identify independent risk factors for complications, OS, and DFS. Kaplan–Meier survival curves and log-rank tests compared survival between groups. Nomograms were constructed using R software, and calibration curves assessed model accuracy. Decision curve analysis evaluated the clinical value of nomograms. A statistically significant difference was defined as a *P*-value less than 0.05 (*P*  < 0.05).

## 3. Results

### 3.1. General Patient Characteristics

This study included a total of 302 patients diagnosed with CRC. Their ages ranged from 27 to 88 years, with a median age of 66 (59, 71.25) years. Of these patients, 170 (56.3%) were male and 132 (43.7%) were female. Preoperatively, 76 patients (25.2%) had comorbid hypertension, 40 patients (13.2%) had diabetes, and 60 patients (19.9%) had a history of abdominal surgery. The body mass index (BMI) ranged from 15.63 to 32.91 kg/m^2^, with an average BMI of 22.51 ± 2.89 kg/m^2^. Preoperative hemoglobin levels ranged from 53 to 162 g/L, with a median level of 113 (100, 127) g/L. A total of 154 patients (51.0%) had a CEA level ≥5 ng/mL. Surgical duration ranged from 97 to 221 min, with a median duration of 155 (149, 170) min. Seventy patients (23.2%) underwent preventive ostomy creation, 74 (24.5%) experienced intraoperative bleeding ≥100 mL, and 11 (3.6%) required blood transfusion. Furthermore, 141 patients (46.7%) had a tumor maximum diameter ≥5 cm, and 208 patients (68.9%) exhibited high/medium tumor differentiation, while 94 patients (31.1%) had poor/undifferentiated tumors. The TNM stage revealed 170 patients (56.3%) at Stages I–II and 132 patients (43.7%) at Stage III.

### 3.2. Preoperative NPRI and Its Impact on Short-Term Complications in Patients Undergoing Laparoscopic Radical Surgery for CRC

#### 3.2.1. Determination of the Optimal NPRI Cutoff for Postoperative Complications in CRC Patients

**Figure 1:**
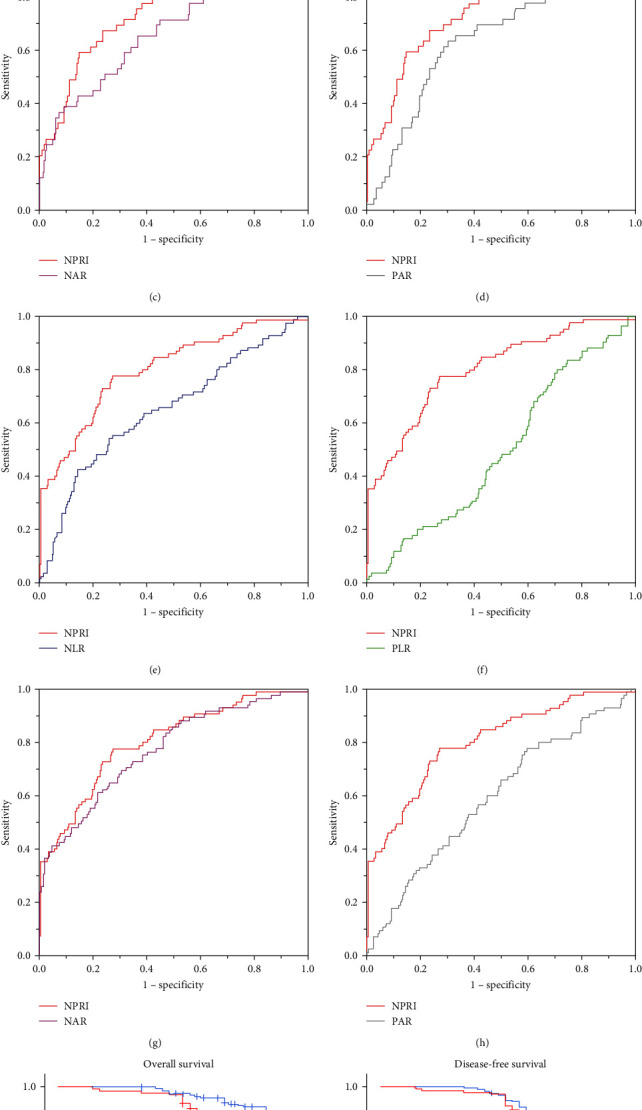
(a–d) ROC curves predicting the occurrence of complications for preoperative NPRI, NLR, PLR, NAR, and PAR; (e–h) ROC curves assessing patient survival time for preoperative NPRI, NLR, PLR, NAR, and PAR; survival curves for OS (i) and DFS (j) based on different NPRI levels.

In this study, preoperative NPRI, NLR, PLR, NAR, and PAR values were calculated for the 302 patients with CRC, and their ability to predict postoperative complications was assessed. ROC curves were constructed to evaluate the predictive performance of NPRI and other indices (see Figure 1(a)–1(d)). The results of the ROC curves are presented, indicating that NPRI (AUC = 0.774), NLR (AUC = 0.606), NAR (AUC = 0.524), and PAR (AUC = 0.649) were all effective in predicting postoperative complications in patients undergoing laparoscopic CRC surgery. Notably, the newly established NPRI demonstrated the highest area under the curve (AUC), signifying superior predictive accuracy.

Based on the ROC curve results, the maximal Youden's index was calculated for NPRI, NLR, NAR, and PAR. The maximal Youden's index values for NPRI, NLR, NAR, and PAR were 0.446, 0.221, 0.297, and 0.333, respectively. The corresponding optimal cutoff values were 3.94 × 10^−2^, 2.54, 17.96 × 10^−2^, and 5.17.

#### 3.2.2. Relationship between Preoperative NPRI and Clinical Characteristics of CRC Patients

Patients were categorized into high and low NPRI groups. Statistically significant differences (*P*  < 0.005) were observed in age, preoperative hemoglobin levels, surgical duration, tumor maximum diameter, and TNM stage between the groups, while no significant disparities were found in other variables. Detailed results can be found in Table S1.

#### 3.2.3. Analysis of Risk Factors for Postoperative Complications in CRC Patients

In this study, a cohort of 302 patients undergoing colorectal surgery was stratified into two groups based on the presence or absence of recent postoperative complications: the noncomplication group and the complication group. Comprehensive statistical analyses were performed to compare general clinical data between these groups.

The results of univariate analysis demonstrated statistically significant differences (all *P*  < 0.005) in various factors, including a history of abdominal surgery, intraoperative bleeding, surgical duration, CEA levels, tumor location, TNM stage, NPRI, NLR, NAR, and PAR. Factors exhibiting significance (*P*  < 0.05) in the univariate analysis were subsequently incorporated into the multivariate analysis. The outcomes of the multivariate analysis identified a history of abdominal surgery, surgical duration, CEA levels, tumor location, and NPRI as independent risk factors influencing the occurrence of short-term postoperative complications in patients undergoing laparoscopic CRC surgery (all *P*  < 0.005). Detailed findings are provided in Tables 1 and 2.

#### 3.2.4. Preoperative NPRI Value and Prediction of Complications in Patients with CRC

In accordance with the Clavien–Dindo classification system, postoperative complications in patients were refined and categorized. Among the 302 patients included in this study, 49 individuals experienced various degrees of postoperative complications, encompassing 13 distinct types. There were 27 cases of Grade I complications, 11 cases of Grade II complications, 6 cases of Grade IIIa complications, 9 cases of Grade IIIb complications, and 6 cases of Grade IV complications, totaling 59 cases (see Table S2). It is important to note that the count of patients with complications does not align with the number of complication cases, as some patients experienced multiple complications.

Within the low NPRI group comprising 236 patients, 20 individuals developed postoperative complications, representing 8.5% of the cohort. In the high NPRI group of 66 patients, 29 patients experienced postoperative complications, accounting for 43.9% of this group. Complications of Grade I and Grade II were collectively categorized as common complications, with 18 cases in the low NPRI group and 20 cases in the high NPRI group. Complications of Grade IIIa, Grade IIIb, and Grade IV were classified as severe complications, comprising 7 cases in the low NPRI group and 14 cases in the high NPRI group. Notably, the *P*-values for these comparisons were all less than 0.001, indicating statistical significance (see Table 3).

### 3.3. Preoperative NPRI Impact on Long-Term Prognosis in Patients Undergoing Laparoscopic Radical Surgery for CRC

#### 3.3.1. Determination of Optimal NPRI Threshold for Long-Term Prognosis in CRC Patients

To assess the impact of preoperative NPRI on long-term prognosis in CRC patients, we calculated NPRI, NLR, PLR, NAR, and PAR for 302 patients and plotted ROC curves, using patient survival status as the state variable (Figure 1(e)−1(h)). The ROC curve results demonstrated the ability of NPRI (AUC = 0.802), NLR (AUC = 0.651), PLR (AUC = 0.496), NAR (AUC = 0.771), and PAR (AUC = 0.594) to predict long-term outcomes following laparoscopic radical surgery for CRC. Notably, the newly established NPRI exhibited the highest AUC, indicating superior predictive accuracy.

Based on the ROC curve results, we determined the maximum Youden's index for NPRI, NLR, NAR, and PAR, with values of 0.504, 0.283, 0.395, and 0.182, respectively. The corresponding optimal cutoff values were identified as 2.87 × 10^−2^, 3.77, 13.88 × 10^−2^, and 4.07.

#### 3.3.2. Relationship between Preoperative NPRI and Clinical Characteristics of CRC Patients

Statistical analysis revealed significant differences (*P* < 0.005) in age, preoperative hemoglobin levels, surgical duration, and TNM stage between high and low NPRI groups, while no significant disparities were found in other variables (see Table S3).

#### 3.3.3. Univariate and Multivariate Cox Regression Analysis of OS in Postoperative CRC Patients

Factors influencing the OS of patients undergoing laparoscopic radical surgery for CRC were analyzed, as indicated in Table 4. Univariate analysis revealed that patient age, history of prior abdominal surgery, surgical duration, tumor maximum diameter, tumor differentiation, TNM stage, NPRI, NLR, NAR, and PAR were closely associated with OS (all *P*  < 0.005). Further multivariate analysis demonstrated that the absence of prior abdominal surgery history, tumor maximum diameter <5 cm, high/medium tumor differentiation, and NPRI <2.87 × 10^−2^ were associated with better OS (all *P* < 0.05).

#### 3.3.4. Univariate and Multivariate Cox Regression Analysis of DFS in Postoperative CRC Patients

Factors affecting DFS in patients undergoing laparoscopic radical surgery for CRC were analyzed, as presented in Table 5. Univariate analysis showed that patient age, hypertension comorbidity, prior abdominal surgery history, surgical duration, tumor maximum diameter, tumor differentiation, TNM stage, NPRI, NLR, and NAR were closely related to DFS (all *P* < 0.005). Subsequent multivariate analysis indicated that the absence of prior abdominal surgery history, tumor maximum diameter <5 cm, high/medium tumor differentiation, TNM Stages I–II, and NPRI < 2.87 × 10^−2^ were associated with better DFS (all *P* < 0.05).

#### 3.3.5. Relationship between NPRI Levels and OS and DFS in Patients

Patients were categorized into high and low NPRI groups based on the optimal NPRI threshold. Among the high NPRI group, 66 patients experienced postoperative mortality, while the low NPRI group had 19 patients with postoperative mortality, resulting in survival rates of 47.20% and 89.27%, respectively. The OS rate was 71.85%. The Kaplan–Meier curve for OS (Figure 1(i)) showed a significantly higher 5-year OS for CRC patients in the low NPRI group compared to the high NPRI group, with a statistically significant difference (*χ*^2^ = 63.520, *P* < 0.001). In the high NPRI group, 55 patients experienced no recurrence, while the low NPRI group had 124 patients with no recurrence or metastasis, resulting in DFS rates of 44.00% and 70.06%, respectively. The overall DFS rate was 59.27%. The Kaplan–Meier curve for DFS (Figure 1(j)) demonstrated a significantly higher 5-year DFS for CRC patients in the low NPRI group compared to the high NPRI group, with a statistically significant difference (*χ*^2^ = 23.512, *P* < 0.001).

### 3.4. Construction and Validation of Prognostic Nomograms for Patients Undergoing Laparoscopic Radical Resection of CRC

**Figure 2:**
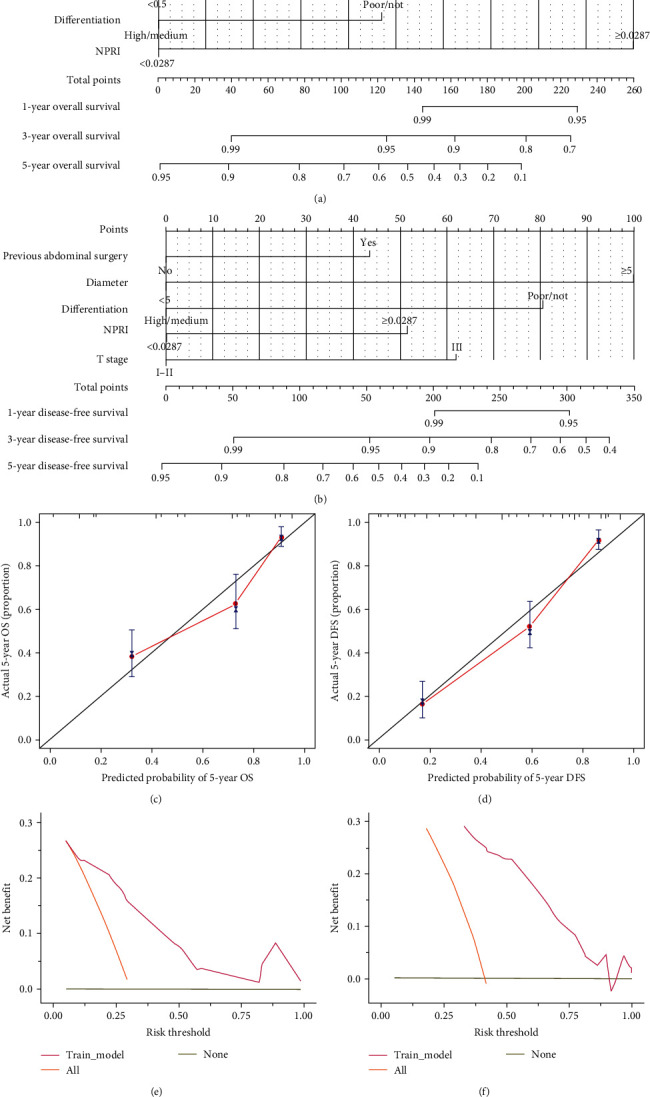
Nomograms predicting the probabilities of OS (a) and DFS (b) following laparoscopic radical surgery for CRC; calibration curves for 5-year OS (c) and DFS (d) predicted by the nomogram model after laparoscopic radical surgery for CRC; decision curve analysis for 5-year OS (e) and DFS (f) predicted by the nomogram model after laparoscopic radical surgery for CRC.

Based on the results of the Cox proportional hazards model, we constructed a nomogram for OS using data from 302 patients who underwent laparoscopic radical resection for CRC to predict the 1-, 3-, and 5-year survival rates of CRC patients after surgery (as shown in Figure 2(a)). This nomogram included four indicators: history of abdominal surgery, tumor maximum diameter, NPRI, and tumor differentiation. Patients with a history of abdominal surgery, tumor maximum diameter ≥5 cm, NPRI ≥ 2.87 × 10^−2^, and low/undifferentiated tumor differentiation had an increased risk of poor prognosis.

In DFS survival analysis, five indicators, namely, history of abdominal surgery, tumor maximum diameter, tumor differentiation, TNM stage, and NPRI, were independently associated with the prognosis of CRC patients. Consequently, we utilized these prognostic factors to construct the DFS nomogram for predicting the DFS of CRC patients at 1-, 3-, and 5-years postoperatively. The nomogram depicted in Figure 2(b) elucidates that a history of abdominal surgery, tumor maximum diameter ≥5 cm, NPRI ≥2.87 × 10^−2^, low/undifferentiated tumor differentiation, and advanced Stage III TNM classification confer an elevated risk of unfavorable prognosis. The C-index for the OS and DFS nomograms was 0.841 (95% CI: 0.727–0.955) and 0.845 (95% CI: 0.707–0.983), respectively. Calibration curves for 5-year OS and DFS demonstrated a high degree of consistency between predicted survival probabilities and observed outcomes (as shown in Figures 2(c) and 2(d)). Furthermore, decision curve analysis (DCA) for 5-year OS and DFS of the nomogram model (as depicted in Figures 2(e) and 2(f)) further confirmed the clinical utility of the nomogram. These results indicate that the nomogram possesses a high degree of accuracy in predicting the prognosis of CRC patients.

## 4. Discussion

Previous studies have mainly investigated the prognostic value of conventional clinical markers in CRC, while their predictive value for short-term complications has been largely overlooked. In our study, we performed ROC curve analysis to compare the predictive ability of NPRI, NLR, PLR, and NAR for postoperative complications in CRC patients who underwent laparoscopic radical resection. We found that NPRI had the best performance among the four markers. Logistic regression analysis revealed that previous abdominal surgery, longer operation time, elevated CEA levels, tumor location in the rectum, and higher NPRI values were independent risk factors for postoperative complications in CRC patients. This further confirms the clinical utility of NPRI in assessing the postoperative risk of patients. We also observed that the incidence of severe complications (Grade III or above) in the high NPRI group was 21.2%, significantly higher than 3.0% in the low NPRI group. This suggests that NPRI can not only predict the occurrence of postoperative complications in CRC patients but also provide a detailed evaluation and stratification for these patients.

Recent studies have reported that NAR is an independent risk factor for postoperative complications in CRC patients, with an optimal cutoff value of 16.50 × 10^−2^ [23]. However, this study did not specify the type of surgery used. In contrast, our study confirmed that NAR could predict the occurrence of postoperative complications, with an optimal cutoff value of 17.96 × 10^−2^. However, NAR was not an independent risk factor for postoperative complications in CRC patients. Moreover, existing research suggests that patients with a history of previous abdominal surgery can still achieve a high success rate with laparoscopic radical resection for CRC, and their previous abdominal surgery history does not affect their recent outcomes [24]. Our research results are different from this, and we speculate that the possible reason is that patients with previous abdominal surgery have different degrees of adhesion in the abdominal cavity, which increases the difficulty of laparoscopic radical resection for CRC, prolongs the operation time and bleeding, and thus increases the probability of postoperative complications such as bleeding and intestinal obstruction, affecting the prognosis of patients [25].

In this prognostic study, we evaluated the performance of five novel biomarkers, namely, NPRI, NLR, NAR, PLR, and PAR, in predicting the long-term outcomes of CRC patients who underwent laparoscopic surgery. We demonstrated that NPRI had the highest accuracy among the five biomarkers. By using the ROC curve analysis, we determined the optimal cutoff value of NPRI to be 2.87 × 10^−2^. Based on this value, we stratified the patients into high and low NPRI groups and found significant associations between NPRI and age, operation time, and TNM stage. The results of univariate and multivariate Cox regression analyses indicated that NPRI ≥ 2.87 × 10^−2^ was an independent risk factor for shorter OS and DFS in CRC patients. Furthermore, we developed a nomogram model to visualize the impact of multiple factors on the prognosis of patients who received laparoscopic radical resection for CRC. The nomogram showed good predictive ability for OS and DFS, and the internal validation cohort confirmed the high predictive accuracy of our prognostic nomogram, suggesting its clinical utility in estimating the long-term outcomes of CR C patients who underwent laparoscopic surgery.

NLR, NAR, PLR, and PAR are effective biomarkers for predicting the prognosis of various types of cancers. Proctor et al. [26] reported that NLR was an important surrogate marker for OS and cancer-specific survival (CSS) in CRC patients. Kim et al. [27] proposed that high PLR (≥160) and high NLR (≥3.0) were independent predictors of poor OS and DFS in Stages III and IV CRC patients, but not in Stages I and II patients. Xie et al. [23] found that NAR had a good predictive role for the postoperative prognosis of CRC patients. Li et al. [28] considered that PAR was a reliable indicator for predicting the short-term survival outcome of critically ill CRC patients. Our study results showed that NPRI, NLR, NAR, and PAR could well predict the short-term and long-term prognosis of patients undergoing laparoscopic radical resection for CRC, but NLR, NAR, and PAR were not independent risk factors for postoperative prognosis in CRC patients. The possible reason for this result may be that the patient population participating in each study was different (patients who underwent radical resection, metastatic CRC patients, CRC with peritoneal metastasis, etc.). Our study found that tumor diameter ≥5 cm and the tumor differentiation degree of poor/undifferentiated patients had poorer OS and DFS; TNM Stages I–II patients had longer DFS, which was consistent with some literature reports [29, 30].

Inflammation and nutritional status are important factors affecting tumor development and clinical outcomes. Although various biomarkers have been widely used to reflect the nutritional and inflammatory status of cancer patients, they have established different predictive or prognostic roles in different cancer types and treatment settings. NPRI, as a newly discovered predictor in our study, can comprehensively and sensitively reflect the perioperative immune ability and nutritional status of CRC patients. Previous studies have shown that NPRI can predict the prognosis of radical resection for intrahepatic cholangiocarcinoma [21]. After analyzing 302 patients who underwent laparoscopic radical resection for CRC, we confirmed that NPRI was an important predictive indicator, which was not only related to short-term complications after laparoscopic radical resection for CRC but also closely related to the long-term prognosis of CRC patients.

NPRI is the preoperative ratio of neutrophils to prealbumin, which reflects the balance between inflammation and nutrition. Neutrophils are the main effector cells of inflammation, and their activation can lead to tissue damage and organ dysfunction [31–33]. Prealbumin is a sensitive indicator of nutritional status, and its low level is associated with poor wound healing and increased infection risk [34]. Prealbumin has also been proven to be an effective prognostic predictor for cancer patients, with higher sensitivity than albumin in predicting the nutritional status of patients [35]. A high NPRI value indicates an increased level of neutrophils and a decreased level of prealbumin, which may imply a more intense inflammatory response and a worse nutritional status, and thus a higher risk of postoperative complications and poor long-term outcomes. Therefore, NPRI may be a useful marker to monitor the inflammatory and nutritional status of CRC patients and to guide the prevention and treatment of postoperative complications and improvement of long-term prognosis.

The gut microbiome is considered an important factor in the development and progression of CRC. It influences the host's immune system, including the quantity and function of neutrophils. Deshmukh et al. [36] found that germ-free mice had significantly lower neutrophil counts than conventional mice, and the introduction of gut microbiota into germ-free mice restored their neutrophil counts. Another study by Karmarkar and Rock [37] demonstrated that alterations in the gut microbiota lead to increased neutrophil activation and recruitment, thereby exacerbating inflammatory responses. Several studies suggest that changes in the gut microbiome may influence the absorption and utilization of nutrients and induce systemic inflammatory reactions, thereby indirectly altering albumin levels [38, 39]. However, which specific microbial communities or species are associated with prealbumin levels still requires further experimental research to elucidate. It can be inferred that the NPRI not only reflects the patient's inflammatory and nutritional status but may also indirectly reflect changes in the gut microbiome, potentially impacting the prognosis of CRC patients.

Our study has several limitations. First, as it was retrospective, bias may be inherent. Second, being a single-center study with a limited number of patients, generalizability may be restricted. Third, the optimal cutoff values of NLR, NAR, and PAR in our study differed from those in other studies, possibly due to variations in the study population, treatment strategies, and perioperative management. Consequently, our results warrant validation through future multicenter, large-sample, prospective studies. Furthermore, our study did not include CRP and IL-6, two crucial inflammatory markers, for comparative analysis. Therefore, additional research is required to explore additional hematological markers or alternative.

In conclusion, our study suggests that preoperative NPRI can serve as a predictive marker for short-term complications and long-term outcomes in patients undergoing laparoscopic radical resection for CRC. Due to its low cost, easy availability, and high predictive value, NPRI may be a reliable marker to help doctors accurately assess the postoperative complication risk and prognosis and take necessary treatment measures for high-risk patients to reduce the occurrence of complications and prolong the survival time of patients.

## Figures and Tables

**Table 1:** Univariate analysis of risk factors for short-term postoperative complications in CRC.

Variables	Complication group (*n* = 49)	Noncomplication group (*n* = 253)	*t*/*z*/*χ*^2^	*P*
Gender	—	—	0.199	0.656
Male	29	141	—	—
Female	20	112	—	—
Age (years)	66 (61, 73)	65 (58.5, 71)	−1.146	0.252
BMI (kg/m^2^)	22.49 ± 3.09	22.52 ± 2.86	0.061	0.952
Preoperative hemoglobin (g/L)	110.90 ± 22.36	112.01 ± 21.92	0.323	0.747
Hypertension	—	—	0.058	0.81
Yes	13	63	—	—
No	36	190	—	—
Diabetes	—	—	2.612	0.106
Yes	10	30	—	—
No	39	223	—	—
History of abdominal surgery	—	—	4.241	0.039 ^*∗*^
Yes	15	45	—	—
No	34	208	—	—
Intraoperative bleeding (mL)	—	—	4.73	0.030 ^*∗*^
≥100	18	56	—	—
<100	31	197	—	—
Intraoperative transfusion	—	—	1.025	0.311
Yes	3	8	—	—
No	46	245	—	—
Surgical duration (min)	179 (158.5, 193.5)	153 (147, 164)	−7.128	<0.001 ^*∗*^
CEA (ng/mL)	—	—	4.795	0.029 ^*∗*^
≥5	32	122	—	—
<5	17	131	—	—
Preventive ostomy	—	—	2.948	0.086
Yes	16	54	—	—
No	33	199	—	—
Tumor maximum diameter (cm)	—	—	0.954	0.329
≥5	26	115	—	—
<5	23	138	—	—
Tumor location	—	—	13.151	<0.001 ^*∗*^
Colon	27	201	—	—
Rectum	22	52	—	—
Tumor differentiation grade	—	—	3.755	0.053
G1–2	28	180	—	—
G3–4	21	73	—	—
TNM stage	—	—	4.29	0.038 ^*∗*^
I–II	21	149	—	—
III	28	104	—	—
NPRI	—	—	47.725	<0.001 ^*∗*^
≥3.94 × 10^−2^	29	37	—	—
<3.94 × 10^−2^	20	216	—	—
NLR	—	—	6.759	0.009 ^*∗*^
≥2.54	36	135	—	—
<2.54	13	118	—	—
PLR	—	—	4.178	0.052
≥74.80	46	208	—	—
<74.80	3	45	—	—
NAR	—	—	30.211	<0.001 ^*∗*^
≥17.96 × 10^−2^	19	23	—	—
<17.96 × 10^−2^	30	230	—	—
PAR	—	—	19.14	<0.001 ^*∗*^
≥5.17	30	73	—	—
<5.17	19	180	—	—

^*∗*^Indicates the statistical significance of all *P* values < 0.05.

**Table 2:** Multivariate analysis of risk factors for short-term postoperative complications in CRC.

Variables	OR	95% CI	*P*
History of abdominal surgery	3.196	1.261–8.099	0.014 ^*∗*^
Intraoperative bleeding	1.141	0.445–2.924	0.784
Surgical duration	1.056	1.032–1.080	<0.001 ^*∗*^
CEA	3.393	1.433–8.033	0.005 ^*∗*^
Tumor location	2.806	1.210–6.507	0.016 ^*∗*^
TNM stage	1.583	0.718–3.490	0.255
NPRI	4.944	1.717–14.238	0.003 ^*∗*^
NLR	0.972	0.400–2.363	0.950
NAR	1.183	0.371–3.774	0.776
PAR	1.793	0.794–4.049	0.160

^*∗*^Indicates the statistical significance of all *P* values < 0.05.

**Table 3:** Comparison of complications in patients with different preoperative NPRI levels.

Complication categories	NPRI		*χ* ^2^	*P*
	Low (<3.94 × 10^−2^) (*n* = 236)	High (≥3.94 × 10^−2^) (*n* = 66)		
Complications	20 (8.5%)	29 (43.9%)	47.725	<0.001 ^*∗*^
Common complications	18	20	24.110	<0.001 ^*∗*^
Severe complications	7	14	26.538	<0.001 ^*∗*^

^*∗*^Complication categories

**Table 4:** Univariate and multivariate analysis of OS in CRC patients after surgery.

Variables	Univariate analysis			Multivariate analysis		
	HR	95% CI	*P*	HR	95% CI	*P*
Gender	0.990	0.645–1.520	0.963	—	—	—
Age	1.025	1.002–1.048	0.036 ^*∗*^	1.006	0.982–1.031	0.614
BMI	1.023	0.950–1.102	0.546	—	—	—
Preoperative hemoglobin	0.998	0.988–1.008	0.732	—	—	—
Hypertension	1.312	0.818–2.105	0.260	—	—	—
Diabetes	0.993	0.527–1.871	0.982	—	—	—
History of abdominal surgery	1.632	1.004–2.652	0.048 ^*∗*^	0.519	0.309–0.874	0.014 ^*∗*^
Intraoperative bleeding	1.287	0.798–2.077	0.301	—	—	—
Intraoperative transfusion	0.895	0.283–2.832	0.850	—	—	—
Surgical duration	1.012	1.001–1.024	0.038 ^*∗*^	0.998	0.987–1.009	0.696
CEA	1.045	0.663–1.600	0.838	—	—	—
Preventive ostomy	0.690	0.395–1.206	0.193	—	—	—
Tumor maximum diameter	0.484	0.315–0.745	0.001 ^*∗*^	2.426	1.535–3.835	<0.001 ^*∗*^
Tumor differentiation grade	2.355	1.533–3.618	<0.001 ^*∗*^	0.392	0.249–0.618	<0.001 ^*∗*^
TNM stage	1.598	1.044–2.446	0.031 ^*∗*^	0.742	0.473–1.164	0.193
NPRI	6.039	3.621–10.072	<0.001 ^*∗*^	0.212	0.111–0.407	<0.001 ^*∗*^
NLR	2.654	1.731–4.067	<0.001 ^*∗*^	0.933	0.568–1.534	0.785
NAR	4.041	2.610–6.256	<0.001 ^*∗*^	0.701	0.400–1.226	0.213
PAR	2.090	1.255–3.483	0.005	0.921	0.512–1.657	0.784

^*∗*^Indicates the statistical significance of all *P* values < 0.05.

**Table 5:** Univariate and multivariate analysis of DFS in CRC patients after surgery.

Variables	Univariate analysis			Multivariate analysis		
	HR	95% CI	*P*	HR	95% CI	*P*
Gender	0.827	0.581–1.178	0.294	—	—	—
Age	1.021	1.002–1.041	0.028 ^*∗*^	1.006	0.986–1.026	0.580
BMI	1.006	0.945–1.070	0.852	—	—	—
Preoperative hemoglobin	0.993	0.985–1.001	0.109	—	—	—
Hypertension	1.552	1.061–2.270	0.023 ^*∗*^	0.939	0.623–1.415	0.763
Diabetes	0.925	0.539–1.588	0.778	—	—	—
History of abdominal surgery	1.717	1.152–2.559	0.008 ^*∗*^	0.459	0.299–0.705	<0.001 ^*∗*^
Intraoperative bleeding	1.330	0.896–1.975	0.157	—	—	—
Intraoperative transfusion	1.034	0.423–2.531	0.941	—	—	—
Surgical duration	1.010	1.000–1.020	0.042	1.004	0.994–1.014	0.445
CEA	0.898	0.630–1.279	0.550	—	—	—
Preventive ostomy	0.821	0.529–1.274	0.379	—	—	—
Tumor maximum diameter	0.278	0.189–0.408	<0.001 ^*∗*^	4.782	3.162–7.232	<0.001 ^*∗*^
Tumor differentiation grade	2.546	1.784–3.634	<0.001 ^*∗*^	0.305	0.207–0.449	<0.001 ^*∗*^
TNM stage	2.331	1.622–3.349	<0.001 ^*∗*^	0.397	0.270–0.586	<0.001 ^*∗*^
NPRI	2.305	1.611–3.298	<0.001 ^*∗*^	0.552	0.338–0.900	0.017 ^*∗*^
NLR	1.712	1.198–2.448	0.003 ^*∗*^	1.060	0.688–1.631	0.792
NAR	2.374	1.665–3.384	<0.001 ^*∗*^	0.781	0.478–1.275	0.323
PAR	1.404	0.955–2.065	0.084	—	—	—

^*∗*^Indicates the statistical significance of all *P* values < 0.05.

## Data Availability

The data that support the findings of this study are available on request from the corresponding author. Survival data and clinical pathology data are available to the public, but personal information will not be disclosed due to privacy or other restrictions.

## Supplementary Materials

Supplementary Materials Table S1: comparison of clinical characteristics between High NPRI and low NPRI Groups in CRC patients. Table S2: statistical analysis of short-term postoperative complications in CRC patients according to Clavien-Dindo classification. Table S3: analysis of clinical and pathological characteristics in CRC patients after surgery based on NPRI levels.
