# Supplementary material for: Impact of Preoperative Neutrophil to Prealbumin Ratio Index (NPRI) on Short-Term Complications and Long-Term Prognosis in Patients Undergoing Laparoscopic Radical Surgery for Colorectal Cancer
Source: Mediators Inflamm. 2024 Apr 26;2024:4465592. doi: 10.1155/2024/4465592 (PMC11068455; doi:10.1155/2024/4465592)
Supplement: Supplementary Materials — Table S1: comparison of clinical characteristics between High NPRI and low NPRI Groups in CRC patients. Table S2: statistical analysis of short-term postoperative complications in CRC patients according to Clavien-Dindo classification. Table S3: analysis of clinical and pathological characteristics in CRC patients after surgery based on NPRI levels. [file 4465592.f1.docx]

**Supplementary table 1. Comparison of Clinical Characteristics between High NPRI and Low NPRI Groups in** CRC **Patients**

| Variables | NPRI≥3.94×10^-2^（n=66） | NPRI＜3.94×10^-2^（n=236） | t / z / χ2 | P |
| --- | --- | --- | --- | --- |
| Gender |  |  | 0.002 | 0.996 |
| Male | 37 | 133 |  |  |
| Female | 29 | 103 |  |  |
| Age (years) | 70（63.75，75） | 65（58，71） | -3.045 | 0.002* |
| BMI（kg/m^2^） | 22.37 ± 3.27 | 22.55 ± 2.78 | 0.44 | 0.66 |
| Preoperative Hemoglobin (g/L) | 108.50（96，119.25） | 115（101，128） | -2.262 | 0.024* |
| Hypertension |  |  | 2.992 | 0.084 |
| Yes | 22 | 54 |  |  |
| No | 44 | 182 |  |  |
| Diabetes |  |  | 0.512 | 0.474 |
| Yes | 7 | 33 |  |  |
| No | 59 | 203 |  |  |
| History of Abdominal Surgery |  |  | 0.151 | 0.698 |
| Yes | 12 | 48 |  |  |
| No | 54 | 188 |  |  |
| Intraoperative Bleeding（ml） |  |  | 0.003 | 0.956 |
| ≥100 | 16 | 58 |  |  |
| <100 | 50 | 178 |  |  |
| Intraoperative Transfusion |  |  | 0.196 | 0.658 |
| Yes | 3 | 8 |  |  |
| No | 63 | 228 |  |  |
| Surgical Duration (min) | 175（154.75，191） | 153（147，164） | -5.976 | <0.001* |
| CEA (ng/ml) |  |  | 1.037 | 0.309 |
| ≥5 | 30 | 124 |  |  |
| <5 | 36 | 112 |  |  |
| Preventive Ostomy |  |  | 0.575 | 0.448 |
| Yes | 13 | 57 |  |  |
| No | 53 | 179 |  |  |
| Tumor Maximum Diameter (cm) |  |  | 6.572 | 0.01* |
| ≥5 | 40 | 101 |  |  |
| <5 | 26 | 135 |  |  |
| Tumor Location |  |  | 1.536 | 0.215 |
| Colon | 46 | 182 |  |  |
| Rectum | 20 | 54 |  |  |
| Tumor Differentiation Grade |  |  | 2.693 | 0.101 |
| G1~2 | 40 | 168 |  |  |
| G3~4 | 26 | 68 |  |  |
| TNM stage |  |  | 4.031 | 0.045* |
| I-II | 30 | 140 |  |  |
| III | 36 | 96 |  |  |

**Supplementary Table 2. Statistical Analysis of Short-Term Postoperative Complications in CRC Patients According to Clavien-Dindo Classification**

| Complications | I | II | IIIa | IIIb | IV | Total |
| --- | --- | --- | --- | --- | --- | --- |
| Stoma prolapse | 0 | 0 | 0 | 1 | 0 | 1 |
| Stoma bleeding | 1 | 0 | 0 | 0 | 0 | 1 |
| Anastomotic fistula | 0 | 2 | 0 | 2 | 0 | 4 |
| Intestinal obstruction | 0 | 2 | 0 | 2 | 0 | 4 |
| Postoperative bleeding | 0 | 0 | 0 | 1 | 0 | 1 |
| Incision infection | 14 | 0 | 4 | 0 | 0 | 18 |
| Intra-abdominal infection | 0 | 2 | 2 | 3 | 1 | 8 |
| Pulmonary infection | 0 | 5 | 0 | 0 | 1 | 6 |
| Urinary system infection | 1 | 0 | 0 | 0 | 0 | 1 |
| Urinary retention | 11 | 0 | 0 | 0 | 0 | 11 |
| Cardiovascular events | 0 | 0 | 0 | 0 | 2 | 2 |
| Respiratory failure | 0 | 0 | 0 | 0 | 1 | 1 |
| Pulmonary embolism | 0 | 0 | 0 | 0 | 1 | 1 |
| Grand Total | 27 | 11 | 6 | 9 | 6 | 59 |

**Supplementary Table 3. Analysis of Clinical and Pathological Characteristics in CRC Patients After Surgery Based on NPRI Levels**

| Variables | NPRI≥2.87×10^-2^ （n=125） | NPRI<2.87×10^-2^  （n=177） | t /z/χ2 | P |
| --- | --- | --- | --- | --- |
| Gender |  |  | 0.385 | 0.535 |
| Male | 73 | 97 |  |  |
| Female | 52 | 80 |  |  |
| Age (years) | 67（63，73） | 64（57，71） | -2.850 | 0.004* |
| BMI（kg/m^2^） | 22.36 ± 2.96 | 22.62 ± 2.84 | 0.513 | 0.474 |
| Preoperative Hemoglobin (g/L) | 108（96，120） | 117（103，130） | -3.772 | <0.001* |
| Hypertension |  |  | 1.496 | 0.221 |
| Yes | 36 | 40 |  |  |
| No | 89 | 137 |  |  |
| Diabetes |  |  | 0.248 | 0.619 |
| Yes | 18 | 22 |  |  |
| No | 107 | 155 |  |  |
| History of Abdominal Surgery |  |  | 0.06 | 0.807 |
| Yes | 24 | 36 |  |  |
| No | 101 | 141 |  |  |
| Intraoperative Bleeding（ml） |  |  | 0.196 | 0.658 |
| ≥100 | 29 | 45 |  |  |
| <100 | 96 | 132 |  |  |
| Intraoperative Transfusion |  |  | 0.078 | 0.78 |
| Yes | 5 | 6 |  |  |
| No | 120 | 171 |  |  |
| Surgical Duration (min) | 166（151.5，185） | 153（147，158） | -5.178 | <0.001* |
| CEA (ng/ml) |  |  | 0.166 | 0.684 |
| ≥5 | 62 | 92 |  |  |
| <5 | 63 | 85 |  |  |
| Preventive Ostomy |  |  | 0.299 | 0.585 |
| Yes | 27 | 43 |  |  |
| No | 98 | 134 |  |  |
| Tumor Maximum Diameter (cm) |  |  | 0.147 | 0.701 |
| ≥5 | 60 | 81 |  |  |
| <5 | 65 | 96 |  |  |
| Tumor Differentiation Grade |  |  | 1.067 | 0.302 |
| G1~2 | 82 | 126 |  |  |
| G3~4 | 43 | 51 |  |  |
| TNM stage |  |  | 7.165 | 0.007* |
| I-II | 59 | 111 |  |  |
| III | 66 | 66 |  |  |
